# Assessing progress towards the elimination of mother-to-child transmission of syphilis in Peru: A national retrospective analysis

**DOI:** 10.1371/journal.pgph.0006951

**Published:** 2026-08-04

**Authors:** Jazmin Qquellon, Ariana Cardenas-Jara, Andrea Castro-Caparó, Gabriel Carrasco-Escobar

**Affiliations:** 1 Health Innovation Laboratory, Institute of Tropical Medicine “Alexander von Humboldt”, Universidad Peruana Cayetano Heredia, Lima, Peru; 2 Doctoral Programme in Health Sciences, Faculty of Medicine, Universidad Nacional Mayor de San Marcos, Lima, Peru; 3 Escuela Profesional de Tecnología Médica, Universidad Privada San Juan Bautista, Lima, Peru; 4 Division of Experimental Medicine, University of California, San Francisco, California, United States of America; University of Toronto, CANADA

## Abstract

To eliminate and control the mother-to-child transmission (MTCT) of syphilis, the World Health Organization (WHO) recommends meeting the following goals: (1) reducing congenital syphilis incidence to ≤ 0.5 cases per 1000 live births, (2) 90% reduction in maternal syphilis incidence between 2018 and 2030, and (3) 95% coverage of syphilis screening during pregnancy. We evaluated the progress in the Peruvian context at the national and departmental levels. A retrospective ecological analysis was conducted using national secondary data on reported cases of congenital and maternal syphilis from 2015 to 2022. For maternal syphilis, the 2022 goal (2) was defined as a 30% reduction from 2018, approximating the 2030 goal. Percentages of syphilis screening and sociodemographic characteristics were obtained from national Peruvian surveys. Temporal trends were examined using segmented regression, while geographic disparities and spatial clustering were assessed using Marascuilo pairwise comparisons and bivariate Local Moran’s I analyses. In 2022, nationwide syphilis screening during pregnancy was 82.6% (95% CI: 80.7%-84.5%), the congenital syphilis rate was 0.82 cases per 1000 live births, and maternal syphilis increased by 53.7% from 2018. At the department level, only one department achieved all goals; 11, either goal (1) or (2); one, both goals (1) and (2); and 12, neither goal. Segmented regression indicated an acceleration in maternal syphilis rates after 2019. Congenital syphilis rates were highest in departments with the greatest proportions of women with lower education and lack of ANC. Spatial analyses identified high–high clusters of congenital and maternal syphilis in northern departments. This study found that none of the WHO’s Syphilis MTCT goals were met in Peru in 2022. Strengthening decentralized prevention strategies, including scaling up syphilis screening, improving access to ANC, and promoting community engagement within national policies, is essential to reversing current trends and eliminating congenital and maternal syphilis by 2030.

## Introduction

Syphilis, caused by the spirochete bacterium *Treponema pallidum*, is one of the most common sexually transmitted infections (STIs), with an estimated 8 million new cases reported globally in 2022 [1]. In the Region of the Americas, the incidence of syphilis among adults aged 15–49 years increased by 30% between 2020 and 2022, while the prevalence of syphilis among pregnant women rose by 28% during the same period. Consequently, an estimated rate of congenital syphilis of 4.98 cases per 1000 live births was reported in 2022 [1,2].

Mother-to-child transmission (MTCT) of syphilis occurs when an infected mother transmits *T. pallidum* to her child, either during pregnancy through the placenta or during childbirth [3]. Without adequate and timely treatment, the newborn can develop congenital syphilis, which may lead to complications such as severe anemia, visual deficits, liver failure, death, and other adverse pregnancy outcomes. Furthermore, untreated patients who survived congenital syphilis may later present symptoms related to tertiary syphilis or neurosyphilis [4].

At the sixty-ninth World Health Assembly in 2016, the World Health Organization (WHO) established global goals to end the STI epidemic by 2030. These goals included a 90% reduction in syphilis incidence worldwide (compared to 2018 levels) and reducing the incidence of congenital syphilis to ≤ 0.5 cases per 1000 live births [5]. To support these efforts, the WHO introduced an official validation process for countries to achieve the elimination and control of the MTCT of syphilis, including additional goals for congenital syphilis elimination, such as achieving ≥ 95% coverage of syphilis screening among pregnant women during antenatal care (ANC), and ensuring that ≥ 95% of maternal syphilis cases received adequate treatment [6].

However, achieving these goals varies across countries due to multiple challenges. In Latin American and the Caribbean (LAC) countries, common barriers included a shortage of healthcare personnel, inefficient follow-up of maternal syphilis cases [7], limited test coverage [8], inadequate improvement in ANC services [9], lower education levels [10], teenage pregnancy [11,12], and poverty [13].

By 2024, only 18 countries worldwide had been officially recognized by the WHO for achieving the elimination of MTCT of syphilis [14]. Cuba was the first country in LAC to receive this validation in 2015 [15]. In addition, previous studies reported that other countries achieved or partially committed to the goals. The Netherlands reached more than 95% of pregnancy syphilis screening between 2009 and 2015 [16], and Switzerland did not report cases of congenital syphilis in the canton of Zurich between 2010 and 2019 [17]. However, even some high-income countries face setbacks, such as New Zealand, which experienced an increase in congenital syphilis incidence in 2018 and 2020, with rates reaching 9.4 cases per 100,000 live births [18]. The strategies adopted by countries like Indonesia, Brazil, and China revealed progress toward eliminating syphilis, but these countries have not yet met the established goals, highlighting the need for continued commitment and investment in effective interventions [19].

In Peru, the National Plan for the Elimination of MTCT of HIV, syphilis, and hepatitis B was launched in 2017 by the Ministry of Health (MINSA) in alignment with the WHO regional initiative for the elimination of MTCT. The plan is implemented through regional health directorates (DIRESAs/GERESAs) and primary health facilities. Key strategies include expanding syphilis screening coverage within ANC, decentralizing the provision of free diagnostic tests and treatment to primary care facilities, delivering sexual and reproductive health education, promoting condom use and safer sexual practices among women of childbearing age and their partners, and strengthening follow-up systems to ensure treatment linkage for infected pregnant women and exposed newborns [20].

Despite this framework, implementation challenges persist, including geographic and cultural barriers to ANC access in remote areas, high healthcare personnel turnover, supply chain deficiencies, persistent stigma within health services, and fragile health information system that contribute to underreporting [20]. These structural vulnerabilities were likely exacerbated during the COVID-19 pandemic (2020–2022), which disrupted antenatal services, reduced screening coverage, interrupted treatment follow-up, and weakened epidemiological surveillance systems, thereby increasing the risk of underdiagnosis and reporting bias [21,22].

Although Peru has adopted a comprehensive national strategy, no recent systematic evaluation of progress toward WHO MTCT elimination targets for syphilis has been conducted at the national or department level. In Brazil, the government certified municipalities for eliminating MTCT of HIV and syphilis in 2022, awarding gold, silver, or bronze certifications based on their progress [23]. Such initiatives can motivate subnational health authorities to strengthen their efforts within local healthcare systems. In this study, we assessed the progress in the Peruvian context at the national and department level towards the following goals in 2022: (1) ≤0.5 cases of syphilis congenital per 1000 live births, (2) a 30% reduction in maternal syphilis incidence between 2018 and 2022 (as an approximation of the full goal period from 2018 to 2030), and (3) 95% coverage of syphilis screening.

## Materials and methods

### Study design

We conducted a retrospective ecological analysis using secondary data from multiple nationally representative sources to assess maternal and congenital syphilis rates, as well as syphilis screening coverage in Peru from 2015 to 2022. These datasets were obtained from publicly accessible national epidemiological reports and contain only anonymized information. All analyses were performed using aggregated data at the departmental and national levels, and no individual-level data were accessed or included in the analysis.

### Study area and population

Peru is a South American country with 34 million inhabitants and is considered a middle- to high-income country, according to the World Bank [24]. Administratively, the country is organized into 25 departments, each of which is further subdivided into provinces; this three-level hierarchy (national – departmental – provincial) is used throughout this study. By demographic area, the departments can be divided into 3 large regions: Coast, Highlands, and Jungle.

We included databases that specifically provided information on live births, pregnant women, or women who had given birth within the last 12 months. According to the Peruvian government, in 2023, there were about 5 million Peruvian women of reproductive age [25]. However, since the number of pregnant women is not available for each department, we calculated the rates using the number of live births per year as the denominator, as this index has previously been used as an approach to estimate pregnant women [25]. Annual live birth data at the national and departmental levels were obtained from the National Registry of Identification and Civil Status (RENIEC).

### Data sources

#### Peruvian National Institutes of Health.

This public repository is managed by the National Center for Epidemiology, Prevention, and Disease Control (CDC) of the Ministry of Health (MoH) of Peru. Surveillance of congenital syphilis in Peru began in 1999, while maternal syphilis surveillance began in 2015, following the approval of the National Technical Regulation for the epidemiological surveillance of maternal and congenital syphilis [26,27]. We collected individual case-level data on maternal and congenital syphilis at provincial and departmental levels from 2015 to 2022. Absolute case counts and corresponding rates per 1000 live births were calculated using annual live birth denominators from RENIEC (Table A in S1 Appendix).

#### Demographic and Health Survey.

The Demographic and Health Survey (DHS) has been prepared by the National Institute of Statistics and Informatics (INEI) since 1986. The main objective of this survey is to provide information regarding the health status of mothers and children [28]. Since 2015, balanced sampling has been applied to collect information, which is carried out throughout the year. In this context, the annual sample allows representative estimates to be made at the national and departmental levels [28]. The DHS employs a complex survey design with stratified, multistage sampling and uses probability weights to ensure population-level representativeness. Using the DHS database, we collected information regarding syphilis screening during pregnancy from 2015 to 2022. ENDES provides nationally and departmentally representative estimates of syphilis coverage through weighted proportions. The 95% confidence intervals (CI) for annual screening coverage were estimated using survey-weighted proportions under the complex sampling design, accounting for primary sampling units, stratification, and sampling weights. Variance estimation was performed using Taylor series linearization with a normal approximation. Screening coverage estimates are reported in Table A in S1 Appendix.

#### National Household Survey.

The National Household Survey (NHS) has been prepared by the INEI since 1995 to monitor key indicators of living conditions [29]. The survey employs a mixed-method approach, covering the entire country through continuous data collection. Its sampling method is probabilistic, stratified, multistage, and independent for each department [29]. The NHS assesses sociodemographic characteristics in different periods, with independent sections on education, health, employment, and income. For this study, we focus on women aged ≥ 15 years old who responded “yes” to the question: *In the last 12 months, have you consulted for: Iron supplementation (pregnant women and children under 3 years of age)?*

### Variables

#### Outcome variables.

At the national and departmental level, we calculated the rates of congenital and maternal syphilis and the percentage of syphilis screening in 2022 to assess progress towards achieving the goals.

Congenital syphilis cases included live births, stillbirths, or spontaneous abortions from a mother with untreated or inadequately treated maternal syphilis; neonates with reactive Rapid Plasma Reagin (RPR) or Venereal Disease Research Laboratory (VDRL) tests with titers four times higher than the mother’s; or those with clinical or radiographic signs of congenital syphilis and a positive result in screening or confirmatory tests [26]. We calculated congenital syphilis incidence rates by dividing the number of new cases by live births per year, multiplied by 1,000.

Maternal syphilis cases were defined by laboratory testing and clinical evaluation: a reactive result in a screening test for syphilis, with or without clinical evidence of primary or secondary syphilis (probable case); and a reactive RPR or VDRL test with titers ≥ 8 dilutions, along with a positive confirmatory test [26]. The rates of maternal syphilis were calculated by dividing the number of new cases by the number of live births per year, multiplied by 1,000.

Syphilis screening is mandatory during pregnancy, according to the National Technical Regulation, to prevent mother-to-child STIs [30]. Syphilis screening is done either with an immunochromatographic test or an RPR test. When a positive result is obtained in the screening, a confirmatory treponemal test is performed, such as Treponema Pallidum Hemagglutination (TPHA) or fluorescent treponemal antibody-absorption test (FTA Abs).

#### Analytical approach to WHO targets.

Since our aim was to assess progress toward achieving the maternal and congenital syphilis elimination goals, we used 2018 as the baseline, as established by the WHO and the Pan American Health Organization (PAHO) in their *Guidance for the Elimination of Syphilis and Congenital Syphilis in the Americas* [5,31].

The congenital syphilis goal was considered achieved if case rates were less than 0.5 cases per 1,000 live births, consistent with WHO and PAHO criteria. In addition, the syphilis screening goal was considered achieved if coverage reached 95% or above.

The maternal syphilis goal aims to reduce overall syphilis incidence by 90% by 2030, using 2018 as the baseline. As the most recent available data corresponded to 2022, we applied a linear interpolation approach. Assuming a constant trajectory towards 2030 target, we expected a decrease in maternal syphilis rates of 30% in 2022 compared to 2018, corresponding to 4 of the 12 years between baseline and goal. Consequently, the goal was considered achieved if maternal syphilis rates in 2022 were at least 30% lower than in 2018.

#### Sociodemographic variables.

We included poverty, education level, and ANC variables based on previous literature. Poverty was classified into two categories: “poor” (which included both extreme poverty and poverty level) and “not poor”. Education level was dichotomized as “lower education” (less than secondary education) and “higher education” (secondary or more). ANC was assessed through prenatal care visits, and was dichotomized as “yes” and “no”. We grouped the departments according to natural regions (Coast, Highlands, and Jungle) to calculate the tertiles of each sociodemographic variable’s provincial (sub-departmental) proportion per year. The tertiles were calculated using the 33rd and 66th percentiles of the survey-weighted province-year estimates where the third tertile represented the provinces within the region with the highest proportion of people living in poverty, lower education, and no ANC, while the first tertile represented the provinces with the lowest proportion of each characteristic. It is important to note that cut-points were estimated separately for each outcome (maternal and congenital syphilis) given differences in their underlying distributions.

### Statistical analysis

The mean maternal and congenital syphilis incidence rates and their standard deviations (SDs) were reported using descriptive statistics, by year and department. Temporal trends were visualized using a dumbbell plots to compare changes across years.

To examine how maternal and congenital syphilis rates varied over time according to sociodemographic vulnerability, estimated mean rates and their 95% CI for each year-by-tertile stratum, both nationally and by natural region were calculated.

To further assess whether geographic disparities in syphilis rates among natural regions persisted across different sociodemographic profiles, provinces were stratified according to the predominant category of poverty, education level and ANC access. Marascuilo pairwise comparisons were then applied within each stratum to test for statistically significant differences in syphilis proportions between regions, while controlling for multiple comparisons.

Segmented regression analysis was performed to identify potential inflection points in temporal trends for congenital syphilis, maternal syphilis, and syphilis screening coverage. Models were fitted using the segmented package in R, with one breakpoint (npsi = 1) estimated for each outcome. The segmented regression approach iteratively estimates the location of the breakpoint and the slopes before and after this point. Statistical significance of slope coefficients was assessed using t-tests, with p < 0.05 considered statistically significant.

Furthermore, to assess spatial autocorrelation, we used GeoDa software version 1.22. Overall spatial autocorrelation was assessed using the Global bivariate Moran’s I statistic, while localized patterns of spatial association were examined using bivariate Local Indicators of Spatial Association (LISA). Statistical significance for both global and local Moran’s I statistics was evaluated using 999 random permutations, and the results were considered significant at p < 0.05. Bivariate LISA cluster maps were generated to characterize significant region-specific spatial relationships between syphilis rates and each covariate using GeoDa v1.22. To visualize the geographic concurrence between syphilis rates and the selected covariates, we generated descriptive bivariate choropleth maps in R v. 4.3.0 using the *biscale*, *ggplot2*, and *sf* packages. Syphilis rates and each covariate were dichotomized at their respective medians and combined into a 2x2 classification scheme representing low-low, low-high, high-low, and high-high combinations across regions.

### Ethical considerations

This study used exclusively publicly accessible, de-identified secondary data from national sources. ENDES and ENAHO surveys are conducted by the Peruvian INEI under national statistical regulations, with voluntary participant enrollment. The Peruvian CDC surveillance data consist of anonymized aggregate case counts without individual identifiers. All analyses were performed using aggregated data at the departmental and national levels; no individual-level identifiable information was accessed. This secondary analysis complies with international ethical standards for the use of publicly available, de-identified data (Declaration of Helsinki, CIOMS guidelines), respecting participant privacy, data anonymization, and purpose-limited analysis. According to institutional guidelines, secondary analyses of fully de-identified, publicly accessible datasets do not require additional ethics committee approval.

## Results

**Fig 1:**
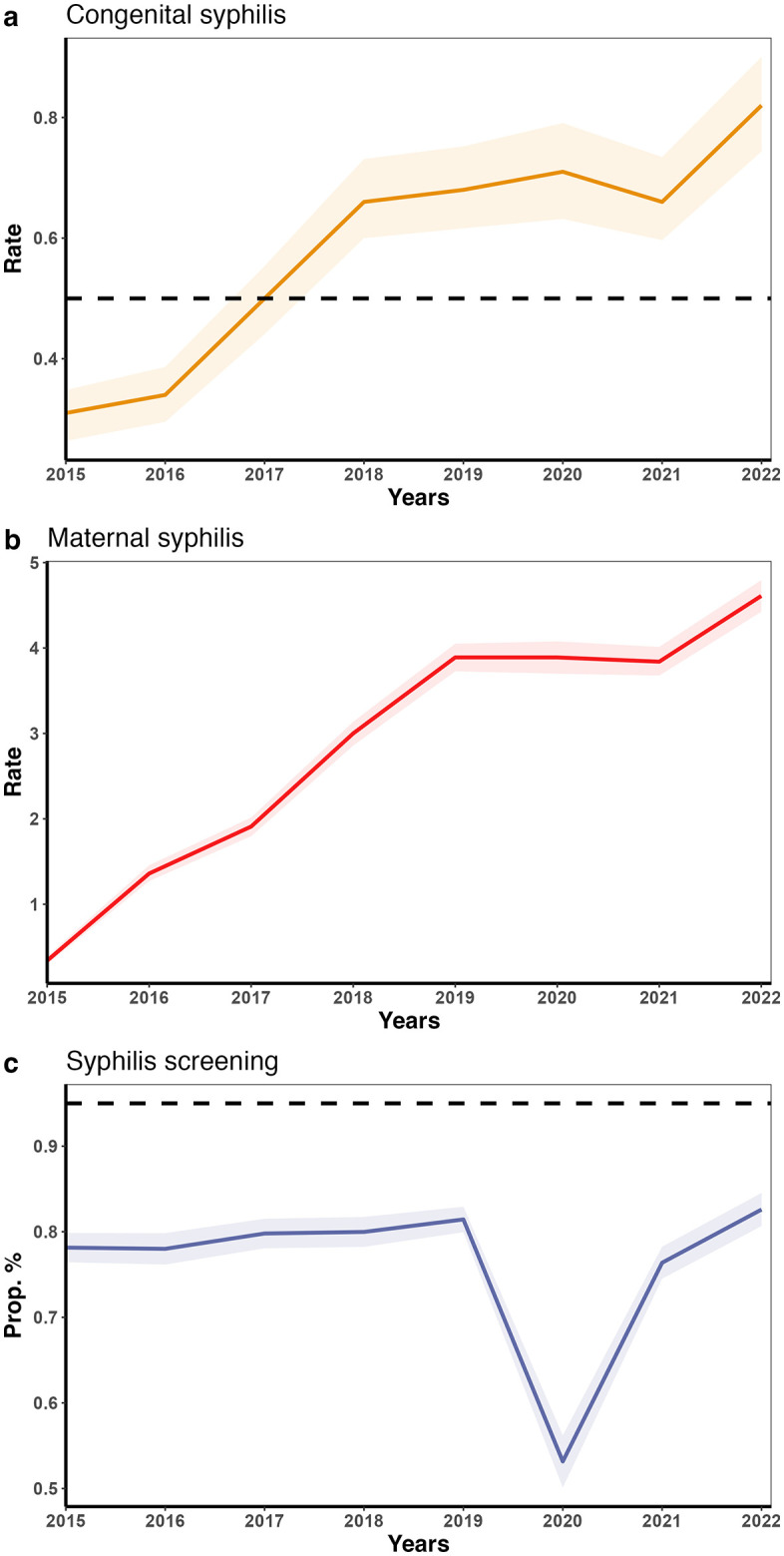
Trends in syphilis in Peru between 2015 and 2022. **a.** Rate of congenital syphilis, with the dashed line indicating the maximum threshold for the goal (≤ 0.5 cases per 1000 live births). **b.** Rate of maternal syphilis. **c.** Proportion of syphilis screening during pregnancy, with the dashed line indicating the minimum screening coverage goal (95%).

Figure 1 shows the national trends in congenital syphilis, maternal syphilis, and syphilis screening between 2015 and 2022. The average congenital syphilis rate was 0.59 cases per 1000 live births (range: 0.31–0.82). Since 2018, the rate has remained above the WHO elimination target of 0.50 cases per 1000 live births, with 2020 and 2022 recording the highest rates at 0.71 and 0.82 cases per 1000 live births, respectively.

For maternal syphilis, the average rate was 2.86 cases per 1000 live births (range: 0.34–4.61), rising steadily since 2018, and peaking at 4.61 cases per 1000 live births in 2022. National syphilis screening coverage averaged 76.2% (range: 53.2%–82.6%). Between 2015 and 2019, coverage increased slightly from 78.1% to 81.4%. However, the positive trend dropped drastically to 53.2% during 2020, due to the large percentage of missing data (35.8%). Importantly, syphilis screening in 2022 showed a positive recovery reaching a percentage of 82.6%. Detailed yearly data are available in Table A in S1 Appendix.

Segmented regression analysis identified potential inflection points for all three outcomes (Fig A in S1 Appendix). For congenital syphilis, a change in trend was observed around 2017–2018 at a rate of approximately 0.41 cases per 1000 live births, with relatively stable or decreasing rates before this threshold and increasing rates thereafter, though slope estimates were not statistically significant (p > 0.05). For maternal syphilis, a breakpoint was observed around 2019 at a rate of approximately 3.2 cases per 1000 live births. Rates increased significantly before this threshold (slope = 0.25, p = 0.044), and more steeply thereafter (slope = 0.52). For syphilis screening, a saturation breakpoint was observed around 2019 at approximately 81% coverage, with no significant association with calendar year before or after this threshold.

At the departmental level, the lowest congenital syphilis rates in 2021–2022 were reported in Madre de Dios and Cusco. Notably, no cases of congenital syphilis were recorded in Madre de Dios during this period. In contrast, the highest rates were observed in Arequipa, Junín, and Moquegua, with rates in 2022 reaching 1.62, 1.31, and 2.23 cases per 1,000 live births, respectively. Maternal syphilis showed a consistent increase from 2015 to 2022 in Madre de Dios, Cusco, Ayacucho, and Ica, with the highest rates reaching 15.49, 6.03, 7.62, and 5.49 cases per 1,000 live births, respectively. Syphilis screening showed an upward trend from 2015 to 2019, followed by a notable drop in 2020 and a slight recovery in 2021–2022. This trend was observed in Amazonas, Huánuco, Ica, Junín, Lima, Loreto, Madre de Dios, Moquegua, San Martín, and Tacna. Additional details were provided in Fig B, Fig C and Fig D in S1 Appendix.

**Fig 2:**
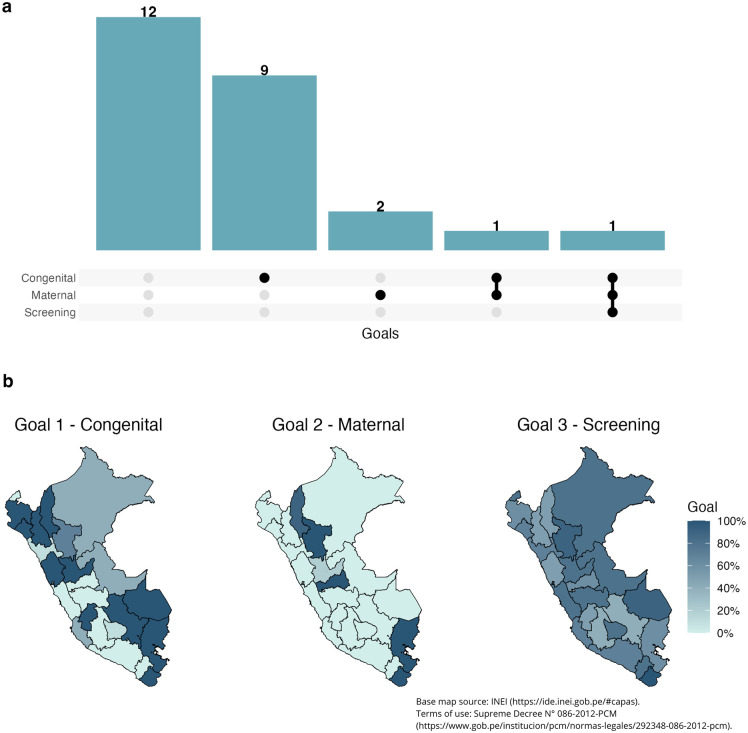
Achievement of syphilis goals by 2022. **a.** Number of departments that met the established goals. **b.** Percentage of goals met at the departmental level. Base map layer: https://ide.inei.gob.pe/#capas.

The achievement of goals in 2022 is shown in Fig 2. At the national level, none of the goals were met: the congenital rate was 0.82 cases per 1000 live births, the maternal syphilis rate increased by 53.7% from 2018, and the syphilis screening coverage was 82.6%. At the department level, only one department achieved all goals; two, maternal syphilis goal only; nine, congenital syphilis goal only; one, both congenital and maternal goals; and 12, neither goal.

Tacna was the only department that accomplished all three goals. In total, 11 departments recorded ≤ 0.5 cases of congenital syphilis per 1000 live births: Piura, Lambayeque, Cajamarca, Ancash, Huánuco, Huancavelica, Amazonas, Madre de Dios, Cusco, Puno, and Tacna. In addition, four departments achieved a reduction of at least 30% in the maternal syphilis rate: Puno, Tacna, Pasco, and San Martín.

**Fig 3:**
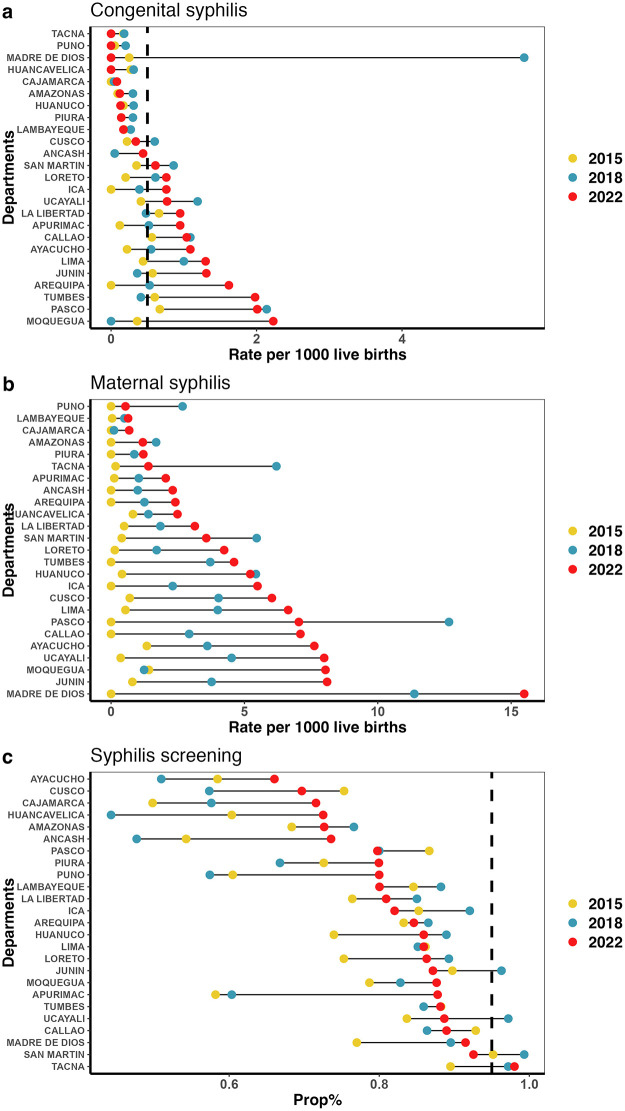
Trends of syphilis in 2015, 2018 and 2022 at the departmental level. **a.** Rate of congenital syphilis at the department level, with the dashed line indicating the maximum threshold for the goal (≤ 0.5 cases per 1000 live births). **b.** Rate of maternal syphilis at the department level. **c.** Proportion of syphilis screening during pregnancy at the department level, with the dashed line indicating the minimum screening coverage goal (95%).

Figure 3 compares the rate of congenital syphilis, maternal syphilis, and syphilis screening in 2015, 2018, and 2022. Regarding congenital syphilis, nine of the 25 departments reported rates below 0.5 cases per 1000 live births across all three years. Most of these departments are located in the northern and central regions of Peru. Conversely, eight departments showed a steady increase in congenital syphilis rates, seven of them had rates exceeding 0.5 cases per 1000 live births in both 2018 and 2022 but not in 2015. Most of these departments are located in the southern and central regions of Peru, including Lima and Callao. Interestingly, three out of five departments in the jungle of Peru exhibited an increasing trend in congenital syphilis rates between 2015 and 2018, followed by a decline in 2022. In Madre de Dios, there were no reported cases in 2022.

Concerning the rate of maternal syphilis in 2015, 2018, and 2022, all departments reported their lowest rates (from 0 to 1.42 cases per 1000 live births) in 2015. Between 2018 and 2022, eight departments recorded an increase of more than 100%. Moquegua showed the largest increase, with rates rising from 1.24 to 8.04 cases per 1000 live births. An increase between 50% to < 100% was observed in six departments, with Apurimac showing the largest rise, from 1.04 to 2.05 cases. An increase of less than 50% was observed in five departments, with Cusco reporting the largest increase, from 4.03 to 6.03. In contrast, six departments reduced their maternal syphilis rates during the same period: Puno (-79.9%), Tacna (-77.4%), Pasco (-44.4%), San Martín (-34.6%), Amazonas (-29.6%), and Huánuco (-3.9%).

In 2018, four departmentsーJunín, Tacna, Ucayali, and San Martínーachieved screening coverage of ≥ 95%; however, by 2022, only Tacna maintained this level. Furthermore, Lima and Callao recorded the highest screening percentages in 2022 (85.9% and 89.0%, respectively). Among other regions, coastal departments had coverage ranging from 73.6% to 88.2% (except Tacna, which reached 98.0%), highland departments from 66.0% to 87.8%, and jungle departments from 72.7% to 92.6%.

**Fig 4:**
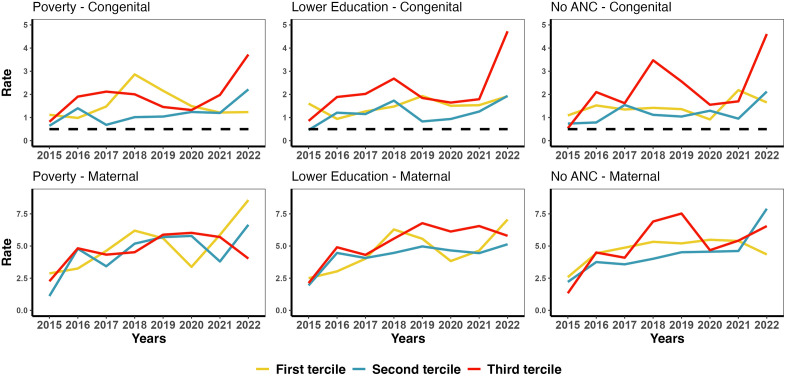
Trends of average syphilis rates for each sociodemographic characteristic at the national level. Each panel shows trends in congenital or maternal syphilis rates from 2015 to 2022, stratified by terciles of sociodemographic characteristics: poverty, lower education, and lack of antenatal care (ANC). Top row: Congenital syphilis rates by sociodemographic characteristics; the dashed line indicates the national goal for congenital syphilis. Bottom row: Maternal syphilis by sociodemographic characteristic. Yellow line: First tercile (lowest burden). Blue line: Second tercile (moderate burden). Red line: Third tercile (highest burden).

Figure 4 illustrates the trends in average syphilis rates stratified of poverty, lower education, and lack of ANC. Maternal syphilis rates showed similar trends across tertiles, while congenital syphilis rates were consistently higher in the third tertile, particularly for lower education and no ANC. Marascuilo pairwise comparisons across natural regions stratified by modal sociodemographic characteristics revealed significant geographic disparities (Table B in S1 Appendix). For maternal syphilis, all regional differences were statistically significant (p < 0.001) across all sociodemographic strata, with the Jungle showing highest proportions (0.00495–0.00521 per live birth), followed by Highlands (0.00339–0.00363) and Coast (0.00188–0.00210). For congenital syphilis, patterns were context-dependent: in departments with lower education, Coast rates were significantly lower than both Highlands and Jungle (p < 0.001), while Highlands and Jungle rates did not differ significantly (p = 0.187). Regarding poverty, Coast rates were significantly lower than both Highlands (p = 0.008) and Jungle (p = 0.001), while Highlands versus Jungle remained non-significant (p = 0.344). Regarding ANC coverage, Coast rates were significantly lower than both Highlands (p = 0.021) and Jungle (p = 0.0035) (Fig E in S1 Appendix).

**Fig 5:**
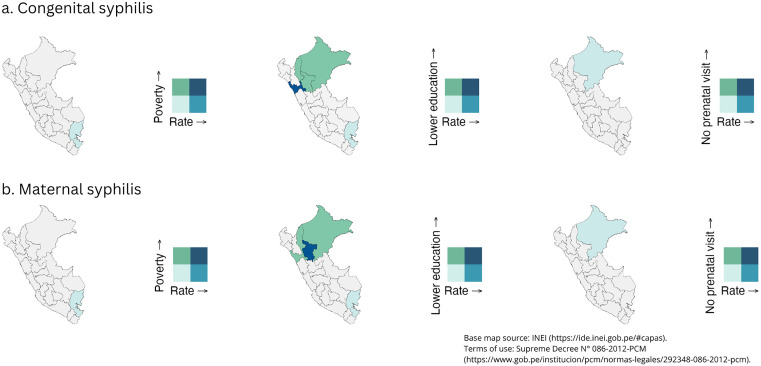
Spatial distribution of syphilis rates and sociodemographic characteristics. Each map shows the co-distribution of syphilis rates (horizontal axis) and sociodemographic characteristics (vertical axis). Sociodemographic characteristics include poverty, lower education, and lack of antenatal care (ANC). Color intensity increases with higher rates of syphilis (left to right) and higher levels of the sociodemographic characteristic (bottom to top). **a.** Congenital syphilis rates and sociodemographic characteristics at the department level. **b.** Maternal syphilis rates and sociodemographic characteristics at the department level. Base map layer: https://ide.inei.gob.pe/#capas.

Figure 5 shows the spatial co-distribution between syphilis rates and sociodemographic characteristics such as poverty, lower education, and lack ANC per department. Global bivariate Moran’s I analyses indicated weak global spatial autocorrelation between congenital syphilis and lower education (I = −0.069), poverty (I = −0.006), and no ANC (I = −0.118), as well as between maternal syphilis and poverty (I = −0.038), lower education (I = −0.117), and no ANC (I = −0.101).

Bivariate Local Moran’s I revealed statistically significant localized clusters. For congenital syphilis, low-low clusters between low congenital syphilis rates with low levels of poverty were identified in the southern central department (Fig 5a). High–high clusters between high congenital syphilis rates co-occurring with high levels of lower education were identified primarily in the northern coastal departments. Furthermore, low–low clusters between low congenital syphilis rates co-occurring with low levels of no ANC were observed in the Jungle.

For maternal syphilis, the clusters were similarly concentrated in comparable departments for each sociodemographic characteristic. High–high clusters between high maternal syphilis rates co-occurring with high levels of lower education were identified primarily in the north-central department (Fig 5b). The co-distribution patterns between syphilis rates and sociodemographic characteristics showed heterogeneous spatial distribution across departments; this trend was consistent across years (Fig F in S1 Appendix).

## Discussion

In this study, we evaluated Peru’s progress towards eliminating MTCT of syphilis at the national and department level using global targets established by WHO: ≤0.5 cases of congenital syphilis per 1000 live births, a 30% reduction in maternal syphilis rates between 2018 and 2022 (one-third of the full period goal), and ≥ 95% coverage of syphilis screening. By 2022, none of these goals were achieved at the national level, with congenital syphilis rates 64% above the threshold, maternal syphilis increasing by 54%, and screening coverage remaining 12% below the target. This represents a critical gap in Peru’s commitment to the WHO STI elimination goals. At the department level, only one met all the goals, while 12 met none, indicating substantial heterogeneity in health system performance. Trend analyses from 2015 to 2022 showed upward trends in congenital and maternal syphilis rates during 2021–2022, coinciding with disruptions to ANC services during COVID-19 pandemic. Additionally, provinces with the highest proportion of women with lower education and lack of ANC had the highest national average rates of congenital syphilis in 2022, suggesting that socioeconomic conditions and barriers to healthcare access may contribute to persistent transmission. These findings underscore the need for quality-focuses, targeted strategies to reduce MTCT of syphilis in Peru.

In 2022, the national congenital syphilis rate was 64% higher than the proposed goal, whereas at the department level, nearly half of the departments met the goal. Exceeding this threshold reflects preventable adverse outcomes, including stillbirth, neonatal death, and long-term disability [4]. According to the Global Health Observatory (GHO), Peru reported a lower rate of 0.65 cases per 1000 live births in 2022 [32]. However, our estimate indicated a slightly higher rate of 0.82 cases per 1000 live births. This discrepancy may be due to differences in data sources. The GHO received Peruvian data from the Health Information System (HIS) of public healthcare centers under the Ministry of Health (MINSA), which represents only one segment of the healthcare system. In contrast, our data was obtained from the Peruvian CDC, which include information from both public and private healthcare establishments [26], and may therefore provide more comprehensive national-levels estimates.

Between 2018 and 2022, the national maternal syphilis rate increased by 53.7%, while at the department level, only four departments achieved ≥30% reductions, and two others reduced their rates without meeting this goal. This sustained increase in maternal syphilis heightens the risk of congenital syphilis, as untreated or late-diagnosed maternal infections directly translate into preventable vertical transmission [4,10]. Surprisingly, the GHO reported a 10.3% decrease in the prevalence of syphilis among Peruvian women attending ANC services during the same period [32]. These discrepancies may be due to differences in data sources and the epidemiological measures used to assess syphilis during pregnancy. Segmented regression revealed a significant acceleration in maternal syphilis rates after 2019, suggesting that service disruptions, diagnostic delays, and missed treatment opportunities—particularly in departments with limited ANC access—may have amplified existing transmission dynamics [4,11,12]. In addition, these trends point to missed opportunities for early intervention and emphasize the need for strengthened partner notification, decentralized and same-day treatment, and improved linkage between diagnosis and care to reverse current trajectories [4,12,33].

According to DHS data from 2022, national screening syphilis coverage was 82.6%, while at the department level only one department reported a screening percentage of 98.0%. Failure to reach the WHO screening target implies missed opportunities for early detection and treatment, limiting the preventive potential of antenatal care to interrupt vertical transmission. The GHO indicated that 21 out of 59 countries met the goal in 2022, with Peru achieving a coverage of 92% [32]. The DHS data may underestimate syphilis screening prevalence due to reporting biases. Trend analyses showed a marked decline in screening coverage in 2020 followed by partial recovery, indicating temporal instability in service delivery rather than sustained improvement. Previous studies have reported comparable national screening syphilis coverage. In Brazil, DHS data reported that 79.8% of pregnant women were tested for syphilis in 2019 [34]. In addition, an ANC sentinel survey in South Africa reported that 96.3% of pregnant women had undergone syphilis testing, with great variation across provinces [35]. At the global and regional levels, the Countdown to 2030 initiative reported that only nine out of 81 countries reached the 95% screening coverage target in 2016–2017 [36], and progress has remained slow in several regions, including sub-Saharan Africa [37], and Pacific Island countries and territories [38].

Our study examined three social characteristics—poverty, education level, and ANC access—in relation to congenital and maternal syphilis. Although global spatial analyses indicated weak overall associations, local clustering revealed significant geographic concentrations of risk. High–high clusters of congenital and maternal syphilis co-occurring with lower education were observed primarily in northern coastal and north-central departments, respectively. Low-low clusters of congenital and maternal syphilis co-occurring with lack of ANC was identified in the Jungle departments. These patterns suggest that social determinants operate through place-specific pathways and that national averages may obscure critical inequities at department level [7,11–13]. Regional comparisons further revealed a persistent Jungle > Highlands > Coast gradient for maternal syphilis regardless of sociodemographic context, suggesting additional structural barriers inherent to the Jungle region beyond social vulnerability. In the Jungle region, geographical remoteness and limited healthcare infrastructure, including shortages of personnel, supplies, and facilities, represent structural barriers that exacerbate inequalities in ANC access [39].

Early and adequate ANC is essential for preventing congenital syphilis, as the lack of ANC was directly associated with higher congenital syphilis rates [9,12]. Strengthening routine screening, ensuring continuity of ANC services, and providing timely partner treatment remain critical to advance toward WHO elimination targets [33,40]. Other factors that affect ANC quality and syphilis risk include young maternal age, lack of a partner, and late ANC initiation [7,11].

Education operates as a structural determinant of access to and use of ANC services [12,13]. Lower education was consistently associated with higher syphilis rates in our analysis, particularly in the Jungle and Highlands, aligning with evidence that women with fewer years of education have lower testing rates [13], inadequate ANC attendance, and higher syphilis risk [11]. Improving ANC coverage must therefore be accompanied by strategies that address educational and literacy barriers to care-seeking.

Poverty did not emerge as a clearly associated factor in this ecological analysis, despite its recognized importance in the broader literature [41]. Global spatial autocorrelation between poverty and syphilis rates was negligible, and the temporal trends in maternal syphilis were similar across poverty tertiles. This may reflect limitations of the ecological design, as the aggregation at the department level likely obscures intradepartmental heterogeneity, and the available poverty measures may not capture the dimensions most relevant to syphilis transmission [42].

Without targeted interventions in high-burden departments, including universal point-of-care testing, ensuring treatment supply, culturally adapted services, and enhanced partner notification, Peru risks widening maternal-child health inequities [3,31]. Future strategies must combine universal ANC access with geographically targeted quality improvement, prioritizing regions where educational and structural barriers most strongly concentrate syphilis risk.

According to our analysis, although we found syphilis testing decreased in 2020, congenital and maternal syphilis rates remained stable and even higher that year, with an upward trend in the following years. This divergence suggests that screening coverage—remaining below the WHO 95% target—represented a missed opportunity for timely diagnosis and treatment during pregnancy, thereby limiting the preventive impact of ANC on vertical transmission. Similar post-pandemic increases in congenital and maternal syphilis have been reported in Spain, the United States, Brazil, Australia, and Japan [43–49]. In several of these settings, apparent declines in 2020 were followed by rebounds in 2021–2022, patterns attributed to disruptions in STI testing and continuity of care that delayed detection and treatment [46]. These findings highlight the importance of protecting essential ANC services during health system shocks and strengthening operational strategies to achieve and maintain screening coverage at or above the 95% target.

We were not exempt from limitations. First, we performed an ecological analysis, which does not allow us to make individual-level inferences. Second, we lacked access to national and departmental-level data on adequate treatment coverage among pregnant women diagnosed with syphilis, including information on treatment timing and regimen. This indicator is one of the core WHO criteria required for validation of congenital syphilis elimination; therefore, its absence limits the ability to fully assess progress towards WHO targets for the MTCT of syphilis [6]. Third, syphilis screening coverage estimates were derived from the DHS survey, which, although designed to be nationally representative, may introduce biases. Fourth, we considered only three sociodemographic variables to geographically evaluate congenital and maternal syphilis trends. These variables were selected based on available NHS data and relevant literature. Additionally, our decision to dichotomize these variables—despite the availability of multiple categories in source data—was driven by the study’s focus on identifying high-vulnerability populations for targeted interventions, though this approach may limit detection of gradient effects or dose-response relationships. Finally, due to the unavailability of public data on the annual number of pregnant women, we used the number of live births per year as a proxy denominator to calculate maternal syphilis rates per 1000 live births. Despite these limitations, this study integrates multiple national data sources to assess in depth syphilis rates and sociodemographic variables through longitudinal and geospatial analyses. This is the first Peruvian study to evaluate progress toward eliminating MTCT of syphilis using transparent and publicly available data.

## Conclusions

This study found that Peru has not achieved any of the WHO targets for preventing mother-to-child transmission of syphilis by 2022. Notably, both congenital and maternal syphilis rates have shown an upward trend in recent years, with a particularly sharp increase in 2022. Delayed screening, missed treatment opportunities, and structural barriers—especially in regions with limited antenatal care and lower education levels—likely contribute to ongoing transmission. Geographic disparities were pronounced, with the Jungle and Highlands showing persistently higher burdens than the Coast.

To achieve the 2030 goals, decentralized strategies must prioritize high-burden regions by expanding point-of-care testing in underserved areas, mandating partner treatment, training community health workers, integrating syphilis and HIV programs, ensuring culturally adapted services, and strengthening awareness campaigns, antenatal care attendance, education, and community engagement. Future efforts should also prioritize improving monitoring of treatment coverage and antenatal care quality to guide resource allocation and support evidence-based policy decisions.

## Supporting information

S1 Appendix Tables A–B and Figs A–F. **Table A.** Trends in congenital syphilis, maternal syphilis, and syphilis screening coverage in Peru, 2015–2022. **Table B.** Marascuilo pairwise comparisons of syphilis proportions across natural regions stratified by sociodemographic characteristics, Peru 2015–2022. **Fig A.** Segmented regression analysis of syphilis trends, Peru 2015–2022. a. Congenital syphilis with inflection point at rate ≈0.41 (around 2017–2018); b. Maternal syphilis with significant breakpoint at rate ≈3.2 (around 2019, p = 0.044); c. Syphilis screening with saturation at ≈81% coverage (around 2019). Red lines represent fitted segmented regression models. **Fig B.** Rates of congenital syphilis per 1000 live births at the department level in Peru, 2015–2022. Base map layer: https://ide.inei.gob.pe/#capas. **Fig C.** Rates of maternal syphilis per 1000 live births at the department level in Peru, 2015–2022. Base map layer: https://ide.inei.gob.pe/#capas. **Fig D.** Percentages of syphilis screening during pregnancy at the department level in Peru, 2015–2022. Base map layer: https://ide.inei.gob.pe/#capas. **Fig E.** Trends of average syphilis rates for each sociodemographic characteristic at the natural region level. Yellow line: First tercile (lowest burden). Blue line: Second tercile (moderate burden). Red line: Third tercile (highest burden). **Fig F.** Spatial distribution of syphilis rates and sociodemographic characteristics in 2015, 2018, and 2022. Congenital syphilis rates and sociodemographic characteristics at the department level (figures a, b, and c). Maternal syphilis rates and sociodemographic characteristics at the department level (figures d, e, and f). Base map layer: https://ide.inei.gob.pe/#capas.
(DOCX)
