# Supplementary material for: Assessing progress towards the elimination of mother-to-child transmission of syphilis in Peru: A national retrospective analysis
Source: PLOS Glob Public Health. 2026 Aug 4;6(8):e0006951. doi: 10.1371/journal.pgph.0006951 (PMC13436696; doi:10.1371/journal.pgph.0006951)
Supplement: Supplementary file 1 [file pgph.0006951.s001.docx]

**Supporting Information**

| Year | Live births (denominator) | Congenital syphilis cases (n) | Congenital syphilis  Rate (95% CI) | Maternal syphilis cases (n) | Maternal syphilis  Rate (95% CI) | Syphilis screening  % (95% CI) | |
| --- | --- | --- | --- | --- | --- | --- | --- |
| 2015 | 652 075 | 199 | 0.31 (0.26 - 0.35) | 224 | 0.34 (0.30 - 0.39) | 78.1 (76.4 - 79.9) | |
| 2016 | 626 259 | 213 | 0.34 (0.30 - 0.39) | 854 | 1.36 (1.27 - 1.46) | 78.0 (76.2 - 79.8) | |
| 2017 | 610 316 | 303 | 0.50 (0.44 - 0.55) | 1 163 | 1.91 (1.80 - 2.02) | 79.8 (78.1 - 81.5) | |
| 2018 | 601 781 | 400 | 0.66 (0.60 - 0.73) | 1 803 | 3.00 (2.86 - 3.14) | 80.0 (78.2 - 81.7) | |
| 2019 | 581 022 | 397 | 0.68 (0.62 - 0.75) | 2 259 | 3.89 (3.73 - 4.05) | 81.4 (80.0 - 82.9) | |
| 2020 | 421 096 | 299 | 0.71 (0.63 - 0.79) | 1 637 | 3.89 (3.70 - 4.08) | 53.2 (50.1 - 56.2) | |
| 2021 | 529 635 | 352 | 0.66 (0.60 - 0.73) | 2 036 | 3.84 (3.68 - 4.01) | 76.4 (74.5 - 78.3) | |
| 2022 | 516 438 | 424 | 0.82 (0.74 - 0.90) | 2 380 | 4.61 (4.42 - 4.79) | 82.6 (80.7 - 84.5) | |
| Rate: cases per 1000 live births | | | | | | |  |
| 95% CI: 95% Confidence Interval | | | | | | |  |
| Annual live birth data were obtained from the National Registry of Identification and Civil Status (RENIEC). | | | | | | |  |
| Note: Syphilis screening coverage estimates are weighted proportions derived from the Demographic and Health Survey (ENDES), which employs complex survey design with probability weights to ensure national and departmental representativeness. These estimates represent population-level screening coverage and do not provide absolute counts of pregnant women screened. | | | | | | |  |

**Table A. Trends in congenital syphilis, maternal syphilis, and syphilis screening coverage in Peru, 2015–2022.**

**Table B. Marascuilo pairwise comparisons of syphilis proportions across natural regions stratified by sociodemographic characteristics, Peru 2015–2022.**

| **Outcome** | **Stratification** | **Comparison** | **Prop 1** | **Prop 2** | **Difference** | **p-value** |
| --- | --- | --- | --- | --- | --- | --- |
| **Maternal syphilis** | Lower education | Coast vs Highlands | 0.00188 | 0.00339 | 0.00151 | **<0.001** |
|  |  | Coast vs Jungle | 0.00188 | 0.00506 | 0.00318 | **<0.001** |
|  |  | Highlands vs Jungle | 0.00339 | 0.00506 | 0.00167 | **<0.001** |
|  | Poor | Coast vs Highlands | 0.00198 | 0.00345 | 0.00147 | **<0.001** |
|  |  | Coast vs Jungle | 0.00198 | 0.00495 | 0.00297 | **<0.001** |
|  |  | Highlands vs Jungle | 0.00345 | 0.00495 | 0.00150 | **<0.001** |
|  | No ANC | Coast vs Highlands | 0.00210 | 0.00363 | 0.00153 | **<0.001** |
|  |  | Coast vs Jungle | 0.00210 | 0.00521 | 0.00311 | **<0.001** |
|  |  | Highlands vs Jungle | 0.00363 | 0.00521 | 0.00158 | **<0.001** |
| **Congenital syphilis** | Lower education | Coast vs Highlands | 0.00044 | 0.00062 | 0.00018 | **<0.001** |
|  |  | Coast vs Jungle | 0.00044 | 0.00070 | 0.00026 | **<0.001** |
|  |  | Highlands vs Jungle | 0.00062 | 0.00070 | 0.00008 | 0.187 |
|  | Poor | Coast vs Highlands | 0.00047 | 0.00062 | 0.00015 | **0.008** |
|  |  | Coast vs Jungle | 0.00047 | 0.00068 | 0.00021 | **0.001** |
|  |  | Highlands vs Jungle | 0.00062 | 0.00068 | 0.00006 | 0.344 |
|  | No ANC | Coast vs Highlands | 0.00053 | 0.00068 | 0.00015 | **0.021** |
|  |  | Coast vs Jungle | 0.00053 | 0.00071 | 0.00018 | **0.035** |
|  |  | Highlands vs Jungle | 0.00068 | 0.00071 | 0.00003 | 0.721 |

**Note:** Marascuilo procedure applied among provinces where each sociodemographic characteristic predominated (modal category). Lower education: < secondary education. Poor: extreme poverty or poverty level. No ANC: no prenatal care visits. P-values from two-proportion z-tests. Significant at α = 0.05.

**Fig A. Segmented regression analysis of syphilis trends, Peru 2015–2022.** a. Congenital syphilis with inflection point at rate ≈0.41 (around 2017–2018); b. Maternal syphilis with significant breakpoint at rate ≈3.2 (around 2019, p = 0.044); c. Syphilis screening with saturation at ≈81% coverage (around 2019). Red lines represent fitted segmented regression models.

**Fig B. Rates of congenital syphilis per 1000 live births at the department level in Peru, 2015–2022.** Base map layer: https://ide.inei.gob.pe/#capas

**Fig C. Rates of maternal syphilis per 1000 live births at the department level in Peru, 2015–2022.** Base map layer: https://ide.inei.gob.pe/#capas

**Fig D. Percentages of syphilis screening during pregnancy at the department level in Peru, 2015–2022.** Base map layer: https://ide.inei.gob.pe/#capas

**Fig E. Trends of average syphilis rates for each sociodemographic characteristic at the natural region level.** Yellow line: First tercile (lowest burden). Blue line: Second tercile (moderate burden). Red line: Third tercile (highest burden).

**Fig F. Spatial distribution of syphilis rates and sociodemographic characteristics in 2015, 2018, and 2022.** Congenital syphilis rates and sociodemographic characteristics at the department level (figures a, b, and c). Maternal syphilis rates and sociodemographic characteristics at the department level (figures d, e, and f). Base map layer: https://ide.inei.gob.pe/#capas
